# Comparison of the clinical benefits for non-small cell lung cancer patients between different volume of pleural lavage fluid following video-assisted thoracoscopic lobectomy and systematic mediastinal lymph node dissection: study protocol for a randomized controlled trial

**DOI:** 10.1186/s13063-020-4146-1

**Published:** 2020-02-27

**Authors:** Jian Zhou, Chengwu Liu, Shulei Man, Mengyuan Lyu, Hu Liao, Nan Chen, Yuhui Cheng, Lunxu Liu

**Affiliations:** 1 Department of Thoracic Surgery, West China Hospital, Sichuan University, No. 37, Guoxue Alley, Chengdu, 610041 Sichuan China; 2 West China School of Medicine, Sichuan University, No. 37, Guoxue Alley, Chengdu, 610041 Sichuan China; 3 Western China Collaborative Innovation Center for Early Diagnosis and Multidisciplinary Therapy of Lung Cancer, Sichuan University, No. 37, Guoxue Alley, Chengdu, 610041 Sichuan China

**Keywords:** Pleural lavage fluid, Pulmonary surgery, Non-small cell lung cancer, Thoracic drainage, Randomized controlled trial

## Abstract

**Background:**

Pleural lavage is regularly performed before closing the chest wall in pulmonary surgeries to prevent pleural implantation of tumor cells and postoperative infection. However, scant data could be found in the literature regarding the optimal regimen for performing pleural lavage. To establish a proper volume of pleural lavage, we herein designed a protocol for a randomized controlled trial.

**Methods:**

A total of 400 participants with non-small cell lung cancer undergoing video-assisted thoracoscopic surgery (VATS) lobectomy and systematic mediastinal lymph node dissection (MLND) will be randomly assigned to one of two groups: group A (500 mL pleural lavage fluid) and group B (3000 mL pleural lavage fluid). The primary outcomes include the levels of leukocytes, neutrophils, and inflammatory factors on the first postoperative day. The secondary outcomes include (i) the levels of leukocytes, neutrophils, and inflammatory factors on the second and third postoperative days; (ii) the incidence of postoperative fever on the first, second, and third postoperative days; (iii) the volumes of chest drainage within the first 3 operative days, the duration of drainage, and postoperative hospitalization; and (iv) the incidence of postoperative complications (incision infection, pain, atelectasis, hemorrhage, etc.) and the incidence of pleural effusion requiring thoracic puncture or drainage within 30 days after surgery. The main content of the analysis includes effectiveness and safety analysis. We will perform subgroup analyses to identify potential influence factors.

**Discussion:**

As far as we know, this will be the first randomized controlled trial to compare the clinical outcomes between different volumes of pleural lavage fluid following VATS and MLND. Findings from this trial will determine the appropriate amount of pleural lavage before chest wall closure.

**Trial registration:**

This study was registered with the Chinese Clinical Trial Registry ( on 17 March 2019. ChiCTR 1900021950).

## Background

In pulmonary surgery, pleural lavage is routinely performed before closing the chest wall, to rinse off residual tumor cells and tissues and ideally prevent pleural implantation of tumor and postoperative infection [1]. Decades ago, it was known that even if there were no obvious malignant pleural effusion or pleural implants, tumor cells could be found in as much a third of postoperative pleural lavage [2]. Since then, accumulating data have indicated the presence of tumor cells in intraoperative pleural lavage as an independent prognostic factor [3–5]. Intraoperative pleural lavage cytology detected before closure could present a higher prognostic value than pleural lavage cytology detected before thoracotomy. Furthermore, it could guide the choice of adjuvant chemotherapy for lung cancer patients after surgery.

Although there is no guideline regarding how pleural lavage should be conducted [6], usually it involves irrigating the thoracic cavity with 0.9% sodium chloride injection varying from 20 to 2000 mL [2, 7–9] at 38~40 °C [10]. However, there are no determinant criteria on the volume of pleural lavage fluid. If the volume of pleural lavage is too small, the residual tumor cells and tissue cannot be washed away, which may result in increased absorption of inflammatory mediators, fevers, and even severe inflammatory reactions [11]. It could affect prognosis and prolong hospitalization [12]. Furthermore, the residual tumor cells may increase the risk of recurrence [13] and metastasis [14–16]. If the volume of pleural lavage is excessive, it will cause waste of resources and prolongation of operation time. Kaneda et al. [9] found that doses of over 500 mL could cause false-negative results of pleural lavage cytology. Considering clinical practice and the literature [7, 8], we decided to test two volumes of pleural lavage: 500 and 3000 mL.

We will prospectively enroll non-small cell lung cancer (NSCLC) patients undergoing video-assisted thoracoscopic surgery (VATS) for lobectomy and systematic mediastinal lymph node dissection (MLND). After enrollment, we will randomly allocate patients to one of two groups: group A (500 mL pleural lavage fluid) or group B (3000 mL pleural lavage fluid). Blood samples will be collected to test for leukocytes, neutrophils, and inflammatory factors. Postoperative complications, the volume of pleural drainage, and length of hospital stay will also be recorded. We aim to compare the clinical benefits for patients with NSCLC between different volumes of pleural lavage fluid following VATS lobectomy and MLND.

## Methods/Design

### Trial design

**Fig. 1:**
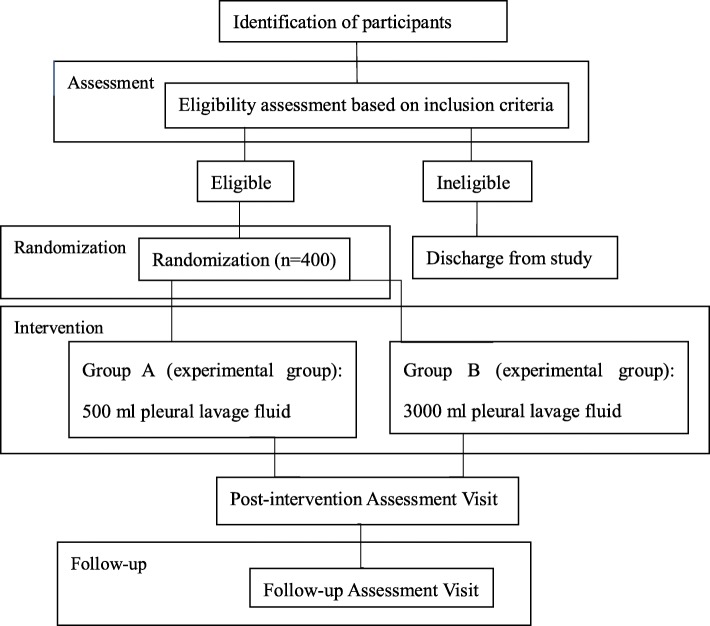
Standard Protocol Items: Recommendation for Interventional Trials (SPIRIT) figure

**Fig. 2:**
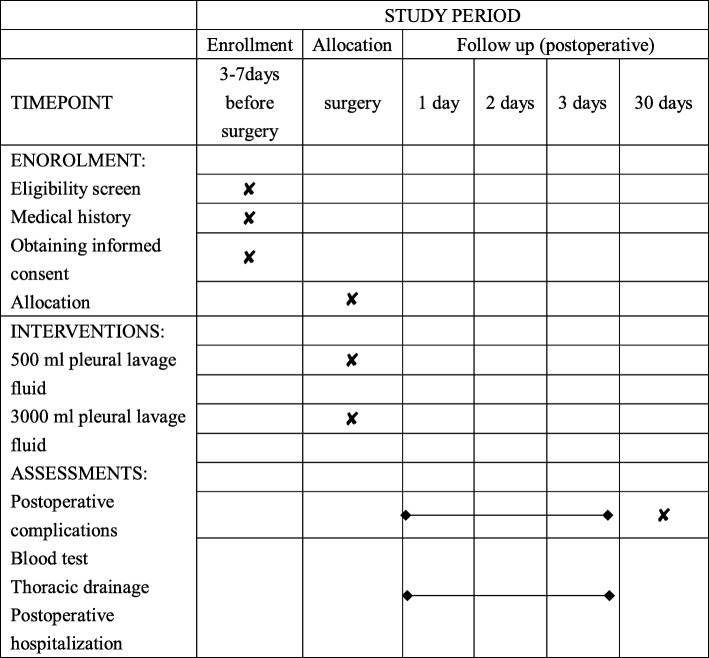
Flowchart for participants’ identification, assessment, enrollment, randomization, intervention, and follow-up

This is a single-blind, single-center, randomized controlled trial (Fig. 1). This study protocol adheres to the Standard Protocol Items: Recommendation for Interventional Trials (SPIRIT) statement. The SPIRIT figure (Fig. 2) summarizes the items of enrollment, intervention, and follow-up. The detailed SPIRIT checklist is also provided (Additional file 1).

### Study objective

This study aims to identify the effects of different volumes of pleural lavage fluid on perioperative outcomes of patients with NSCLC following VATS lobectomy and MLND.

### Study location

This study will be conducted in NSCLC patients undergoing VATS lobectomy and MLND in the Department of Thoracic Surgery, West China Hospital, Sichuan University.

### Recruitment

#### Recruitment of participants

Patients eligible for this trial must comply with all the inclusion criteria and must not meet any exclusion criteria. To achieve adequate enrollment, all surgeons in the thoracic department of the hospital are informed of this trial. Each included patient will sign an informed consent form. The consent form includes (i) the detailed explanation of the study design, including backgrounds and aims of this trial; (ii) the benefits and risks of participating; and (iii) the strategy and compensation for the participants if they experience any harm as a result of trial participation.

#### Inclusion criteria

Patients who meet all the following criteria at the start of the treatment are eligible for this study: (i) patients between 18 and 75 years of age, (ii) patients undergoing planned VATS lobectomy and MLND, (iii) be American Society of Anesthesiologists grade I or II, (iv) essential materials such as clinical staging of lung cancer and medication were complete, (v) confirmed diagnosis of NSCLC through pathological examination after surgery, and (vi) willing to participate after reading and signing an informed consent form.

#### Exclusion criteria

Patients who meet any of the following criteria at the start of treatment are excluded from this study: (i) last smoked fewer than 2 weeks prior to surgery for current smokers, (ii) preoperative hydrothorax of patients was predominant, (iii) patients were pregnant or breastfeeding (females from 18 to 55 years of age should receive a pregnancy test), (iv) patients with preoperative severe mental illness, (v) patients with preoperative gastrointestinal or blood system disease, (vi) patients underwent cardiac ischemia, (vii) patients receiving preoperative radiotherapy or neoadjuvant chemotherapy, (viii) intraoperative accidents such as hemorrhage (>500 mL), conversion to open surgery, or cardiac arrest happened to the patients, and (ix) patients with severe postoperative bleeding or persistent air leakage, which require reoperations.

### Randomization and blinding

Randomization of trial participants will be based on computer-generated random numbers prior to surgery. The random numbers will be printed and placed in consecutively numbered and separate sealed opaque envelopes, which will be opened only when a patient is enrolled and meets all inclusion criteria. The principal doctor (CL) will assign the participant to a group on the basis of the number. The research assistant should receive the notification in a timely fashion and assign patients to their study group strictly as required. This study will be single-blind. The participants will be blinded to the allocation of the participants, whereas the investigators and project manager will be unblinded. If an unexpected emergency occurs, allocation will be disclosed to the investigators, the participant will be withdrawn from this study, and a detailed explanation will be recorded if unblinding happens.

### Sample size

This is the first study that focuses on the effect of different volume of pleural lavage on the clinical outcomes following VATS lobectomy and MLND, and no reference is available to estimate the sample size. We estimated the power on the basis of the Student’s *t* test of the levels of leukocytes on the first postoperative day in each group. We estimate an effect size of 0.5 from our experience. A total of 400 participants will be recruited in this study (200 in each group). Judging from our experience, we set the dropout rate at 10% to account for inability to complete the treatment, data errors, loss to follow-up, and other unanticipated study problems. Given a type I error rate of 5%, this study could provide a power of 99.72% by using G*Power (software version 3.1.3, University of Düsseldorf, Germany).

### Intervention

A total of 400 NSCLC patients who are 18 to 75 years of age and who are undergoing VATS lobectomy and MLND will be recruited in our study on the basis of the inclusion and exclusion criteria. The patients will be divided into two groups:

#### Group A (experimental group): 500 mL pleural lavage fluid

Before closing the chest wall, we will perform careful hemostasis and then flush the thoracic cavity with 500 mL 0.9% sodium chloride injection at 38–40 °C. A 28-F catheter will be indwelled for chest drainage.

#### Group B (experimental group): 3000 mL pleural lavage fluid

We will use 3000 mL 0.9% sodium chloride injection at 38–40 °C to flush the thoracic cavity in this group. All other procedures are the same as those of group A.

### Study dropouts

All recruited participants have the right to quit this study at any time for any reason based on the ethical consideration without any negative effects on their further therapy. Meanwhile, all researchers have the right to terminate the enrollment of any patients at any time within reasonable circumstances. All changes and reasons will be recorded immediately in the case report form (CRF). If the dropout rate is higher than 10%, we will apply multiple imputation to avoid pitfalls involved with listwise deletion of cases. The intention-to-treat principle will be applied to analyze the data.

### Data management

All data recorded in the CRF will be checked twice by two independent researchers. A data management safety committee composed of three independent investigators will be needed. They will supervise the study protocol adherence and participants’ recruitment and confirm that the CRF is correctly completed and consistent with the original data. All data can be acquired only by the study investigators who have signed the confidential disclosure agreement. We do not plan to collect personal information about potential and enrolled participants beyond what is collected during normal hospitalization. After the trial, personally identifiable information will be omitted and placed in a separate database before any data analysis is performed. No participants’ data collected in this trial will be used for other ancillary studies. The adherence to the study protocol, data collection, statistical analysis, and publication issue and related safety issues will be strictly monitored by the institutional ethics committee of West China Hospital, Sichuan University.

### Statistical analysis

The main content of the analysis consists of effectiveness analysis and safety analysis. The analysis of all continuous variables will be presented as mean, standard deviation (SD), median, quartile spacing, and maximum and minimum values. The analysis of all dichotomous variables will be presented as rate, constituent ratio, and hazard ratio. We will use the *t* test and chi-squared test, analysis of variance, and univariate and multivariate logistic regression analysis to describe our data. The factors (*P* <0.15) in univariate analysis will be analyzed in multivariate analysis. All data will be checked twice by two independent statisticians. The two independent statisticians will also be blinded to treatment assignment. We will perform post-hoc subgroup analysis to identify potential significant factors based on age, sex, tumor location, clinical stage of tumor, resection scope, duration of surgery, the volume of intraoperative bleeding, and pathological stage of tumor. Demographics and clinical characteristics of the subjects are summarized as mean ± SD for continuous variables and as number (percentage) for categorical variables. The difference between groups will be considered statistically significant if the *P* value is less than 0.05. All data will be analyzed by using SPSS (software version 25.0; SPSS Inc., Chicago, IL, USA).

### Study organization

#### Data collection and outcomes

We will collect blood samples of patients to test leukocytes, neutrophils, and inflammatory factors. Sample collection will be performed by trained nurses. Samples will be sent to the Department of Laboratory Medicine immediately after collection. The laboratory evaluation will be conducted by technicians, who will be blinded to treatment groups. Laboratory results will be placed in an electronic chart. Specimens will be destroyed and not stored for any ancillary studies. Preoperative data will be collected within 3 days after recruitment. Surgery data will be collected within 2 days after operation. Postoperative data will be collected within 3 days after discharge. For patients discharged home, we will conduct follow-up information by phone calls and these data will be recorded within 3 days after follow-up. If there are any errors or omissions in the electronic chart, the investigator will correct them immediately. The raw data will be marked clearly when revising and will be signed by the investigator with date when the modifications are made. All data can be obtained only by the study researchers who have signed the confidential disclosure agreement.

#### Complications

Some postoperative complications, such as bleeding, pain at the incision site, postoperative air leak, prolonged air leak, and atelectasis, will be treated in accordance with clinical guidelines. During every ward round, conducted at least twice a day, the doctors in charge will solicit the patients’ feedback and perform specific physical examinations to monitor any adverse events. All adverse events will be recorded in a timely fashion in the CRF. Postoperative follow-up will be conducted for all participants. Participants with any serious harm experienced as a result of trial participation will receive adequate compensation.

#### Primary and secondary outcomes

All outcomes will be defined in accordance with two previous studies [17, 18]. The primary outcomes are the levels of leukocytes, neutrophils, and inflammatory factors—interleukin-1β (IL-1β), IL-6, IL-8, IL-2, tumor necrosis factor-alpha (TNF-α), C-reactive protein (CRP), prostaglandin E_2_ (PGE_2_), and 5-hydroxytryptamine (5-HT)—on the first postoperative day. On the first postoperative morning, a trained nurse will collect blood samples and then send samples to test. The mean difference of the levels of leukocytes, neutrophils, and inflammatory factors will be compared between the two groups.

The secondary outcomes are (i) the levels of leukocytes, neutrophils, and inflammatory factors (IL-1β, IL-6, IL-8, IL-2, TNF-α, CRP, PGE_2_, and 5-HT) on the second and third postoperative days; (ii) the incidence of postoperative fever on the first, second, and third postoperative days; (iii) the volumes of chest drainage within the first 3 operative days, the duration of drainage, and postoperative hospitalization; and (iv) the incidence of postoperative complications (incision infection, pain, atelectasis, hemorrhage, etc.) and the incidence of pleural effusion requiring thoracic puncture or drainage within 30 days after surgery.

### Protocol amendments

The current protocol is version 1.0 (September 25, 2018). Any amendment to the protocol that may affect the process of study or the benefits and risks to participants will require the agreement of the ethics committee.

## Discussion

It is essential to flush the thoracic cavity before chest wall closure. However, scant data could be found in the literature. The most frequently used method is to flush the thoracic cavity with 0.9% sodium chloride injection heated nearly to the temperature of the human body at 38~40 °C. No determinant criteria on volume of pleural lavage fluid have been built. If the volume of pleural lavage is too small, the residual tumor cells and tissue cannot be washed away, which may result in increased absorption of inflammatory mediators, fever, and even severe inflammatory reactions and will affect prognosis and prolong hospital stay. Furthermore, the residual tumor cells may increase the risk of recurrence and metastasis. If the volume of pleural lavage is too high, it will cause waste of resources and prolongation of operation time.

The study will enroll 400 NSCLC patients undergoing VATS lobectomy and MLND and divide them into two groups. This study aims to find out whether different volumes of pleural lavage fluid (0.9% sodium chloride injection) have different effects on prognosis of NSCLC patients measuring by some important clinical indices such as the plasma levels of leukocytes, neutrophils, inflammatory factors, and the incidence of fever after operation were observed 1 to 3 days after operation.

However, the study has some limitations. First, it is a single-center trial, which will restrict its generalizability, so a multiple-center large-sample clinical trial is warranted in the future. Second, the anesthesiologist and surgeons in charge of the intraoperative part of the study cannot be blinded to this study group regarding the safety. Third, the hospitalization time may be different across participants, which may bring effects on the prognosis of patients with NSCLC. Fourth, larger patients may need different volumes of pleural lavage.

## Conclusion

This study is the first randomized controlled trial aiming to compare the clinical benefits for NSCLC patients between different volumes of pleural lavage fluid following video-assisted thoracoscopic lobectomy and systematic lymph node dissection. This study may help to develop a standardized procedure of pleural lavage before closing the thoracic cavity in patients undergoing lung cancer surgery.

## Trial status

This study is not yet open for recruitment. This trial was scheduled to begin in July 2019 and to end in July 2021.

## Supplementary information

**Additional file 1.** SPIRIT (Standard Protocol Items: Recommendation for Interventional Trials) 2013 Checklist.

**Additional file 2.** The items from the World Health Organization Trial Registration Data Set.

## Data Availability

The results of this trial will be published in an international peer-reviewed journal and presented at international scientific meetings. No later than three years after the publication of the results of this trial, we will deliver a completely deidentified data set to an appropriate data archive for sharing purposes. Any data request will be sent to the corresponding author and considered carefully.

## Abbreviations

5-HT: 5-hydroxytryptamine
CRF: Case reported form
CRP: C-reactive protein
IL: Interleukin
MLND: Mediastinal lymph node dissection
NSCLC: Non-small cell lung cancer
PGE: Prostaglandin E_2_
SD: Standard deviation
SPIRIT: Standard Protocol Items: Recommendation for Interventional Trials
TNF-α: Tumor necrosis factor-alpha
VATS: Video-assisted thoracoscopic surgery
