# Supplementary material for: Comparison of the clinical benefits for non-small cell lung cancer patients between different volume of pleural lavage fluid following video-assisted thoracoscopic lobectomy and systematic mediastinal lymph node dissection: study protocol for a randomized controlled trial
Source: Trials. 2020 Feb 27;21:232. doi: 10.1186/s13063-020-4146-1 (PMC7047403; doi:10.1186/s13063-020-4146-1)
Supplement: Supplementary file 2 — Additional file 2. The items from the World Health Organization Trial Registration Data Set. [file 13063_2020_4146_MOESM2_ESM.docx]

Additional file 2 The items from the World Health Organization Trial Registration Data Set

| Data category | Information |
| --- | --- |
| Primary registry and trial identifying number | ChiCTR1900021950 |
| Date of registration in primary registry | 17 March 2019 |
| Sponsor | West China Hospital, Sichuan University |
| Contact for public/scientific queries | LL (lunxu_liu@aliyun.com);  JZ (jianzhouscu@foxmail.com);  CL (haibushiwo@163.com) |
| Public title | A prospective, randomized controlled, single-center clinical trial: comparison of the clinical benefits for non-small cell lung cancer (NSCLC) patients between the different volume of pleural lavage fluid following video-assisted thoracoscopic lobectomy and systematic lymph node dissection |
| Scientific title | A prospective, randomized controlled, single-center clinical trial: comparison of the clinical benefits for non-small cell lung cancer (NSCLC) patients between the different volume of pleural lavage fluid following video-assisted thoracoscopic lobectomy and systematic lymph node dissection |
| Countries of recruitment | China |
| Health condition(s) or problem(s) studied | Pulmonary disease |
| Intervention(s) | 500 ml pleural lavage fluid vs. 3000 ml pleural lavage fluid |
| Key inclusion and exclusion criteria | Inclusion criteria:  (i) patients aged between 18 and 75 years; (ii) patients undergoing planned VATS lobectomy and MLND; (iii) be ASA grades I-II; (iv) essential materials were complete such as clinical staging of lung cancer and medication; (v) confirmed diagnosis of NSCLC through pathological examination after surgery; (vi) willing to participate after reading and signing an informed consent form.  Exclusion criteria:  (i) last smoked <2 weeks prior to surgery for current smokers; (ii) preoperative hydrothorax of patients was predominant; (iii) patients were pregnant or breastfeeding women (females aged 18 to 55 should receive pregnancy test); (iv) patients with preoperative severe mental illness; (v) patients with preoperative gastrointestinal or blood system disease; (vi) patients underwent cardiac ischemia; (vii) patients receiving preoperative radiotherapy or neoadjuvant chemotherapy; (viii) intraoperative accidents happened to the patients, such as hemorrhage (>500ml), conversion to open surgery, or cardiac arrest; (ix) patients with severe postoperative bleeding or persistent air leakage, which require reoperations. |
| Study type | Interventional Allocation: randomized; intervention model: parallel assignment; blinding: participants blinding; Primary purpose: treatment |
| Date of first enrolment | Not yet open for recruitment |
| Target sample size | 400 |
| Recruitment status | Not yet open for recruitment |
| Primary outcome(s) | The levels of leukocytes, neutrophils, and inflammatory factors on the first postoperative day. |
| Key secondary outcome | (i) The levels of leukocytes, neutrophils, inflammatory factors on the second and third postoperative day; (ii) the incidence of postoperative fever on the first, second and third postoperative day; (iii) the volumes of chest drainage within the first 3 operative days, the duration of drainage, and postoperative hospitalization; (iv) the incidence of postoperative complications (incision infection, pain, atelectasis, hemorrhage, etc.). |

ChiCTR: Chinses Clinical Trial Registration; NSCLC: non-small cell lung cancer; VATS: video-assisted thoracoscopic surgery; MLND: mediastinal lymph node dissection; ASA: American Society of Anesthesiologists.
